# Mitochondrial Fatty Acid Oxidation Disorders Associated with Short-Chain Enoyl-CoA Hydratase (ECHS1) Deficiency

**DOI:** 10.3390/cells7060046

**Published:** 2018-05-23

**Authors:** Alice J. Sharpe, Matthew McKenzie

**Affiliations:** 1 Department of Biochemistry and Molecular Biology, Monash Biomedicine Discovery Institute, Monash University, 3800 Melbourne, Australia; alice.sharpe@monash.edu; 2 Centre for Innate Immunity and Infectious Diseases, Hudson Institute of Medical Research, 3168 Melbourne, Australia; 3 Department of Molecular and Translational Science, Monash University, 3168 Melbourne, Australia

**Keywords:** mitochondrial disease, fatty acid oxidation, short-chain enoyl-CoA hydratase, ECHS1 deficiency, metabolism, oxidative phosphorylation, OXPHOS

## Abstract

Mitochondrial fatty acid β-oxidation (FAO) is the primary pathway for fatty acid metabolism in humans, performing a key role in liver, heart and skeletal muscle energy homeostasis. FAO is particularly important during times of fasting when glucose supply is limited, providing energy for many organs and tissues, including the heart, liver and brain. Deficiencies in FAO can cause life-threatening metabolic disorders in early childhood that present with liver dysfunction, hypoglycemia, dilated hypertrophic cardiomyopathy and Reye-like Syndrome. Alternatively, FAO defects can also cause ‘milder’ adult-onset disease with exercise-induced myopathy and rhabdomyolysis. Short-chain enoyl-CoA hydratase (ECHS1) is a key FAO enzyme involved in the metabolism of fatty acyl-CoA esters. ECHS1 deficiency (ECHS1D) also causes human disease; however, the clinical manifestation is unlike most other FAO disorders. ECHS1D patients commonly present with Leigh syndrome, a lethal form of subacute necrotizing encephalomyelopathy traditionally associated with defects in oxidative phosphorylation (OXPHOS). In this article, we review the clinical, biochemical and genetic features of the ESHS1D patients described to date, and discuss the significance of the secondary OXPHOS defects associated with ECHS1D and their contribution to overall disease pathogenesis.

## 1. Mitochondrial Metabolism

Mitochondria are the ‘powerhouses’ of the cell, producing 95% of all cellular energy in the form of adenosine triphosphate (ATP) [1]. ATP is expended for essential processes such as growth, reproduction, cell signaling and differentiation [2]. Additionally, mitochondria are involved in other important cellular functions including apoptosis, calcium homeostasis, biosynthesis of heme and iron-sulfur clusters, as well as innate immune responses [2,3].

Under aerobic conditions, mitochondria produce ATP via three key biochemical pathways: the tricarboxylic acid (TCA) cycle, oxidative phosphorylation (OXPHOS) and fatty acid β-oxidation (FAO). Acetyl-coenzyme A (acetyl-CoA) derived from sugars, fats and proteins is oxidized via the TCA cycle to generate the reducing equivalents nicotinamide adenine dinucleotide (NADH) and flavin adenine dinucleotide (FADH_2_) [4]. NADH and FADH_2_ are then oxidized by complex I (NADH: ubiquinone oxidoreductase) and complex II (succinate: ubiquinone oxidoreductase) respectively of the respiratory chain (RC) to drive ATP generation by OXPHOS. The electrons liberated from NADH and FADH_2_ are transferred via ubiquinone to complex III (ubiquinol: ferricytochrome *c* oxidoreductase), then cytochrome *c*, and finally complex IV (cytochrome *c* oxidase) which reduces O_2_ to generate H_2_O. This electron transfer facilitates the pumping of protons out of the mitochondrial matrix by complexes I, III and IV, which establishes an electrochemical membrane potential (ΔΨ_m_) across the inner mitochondrial membrane (IMM) [5]. ΔΨ_m_ drives protons back into the mitochondrial matrix through complex V (F_1_F_0_-ATP synthase), resulting in the phosphorylation of adenosine diphosphate (ADP) to generate ATP [5].

## 2. Fatty Acid β-Oxidation (FAO)

**Figure 1:**
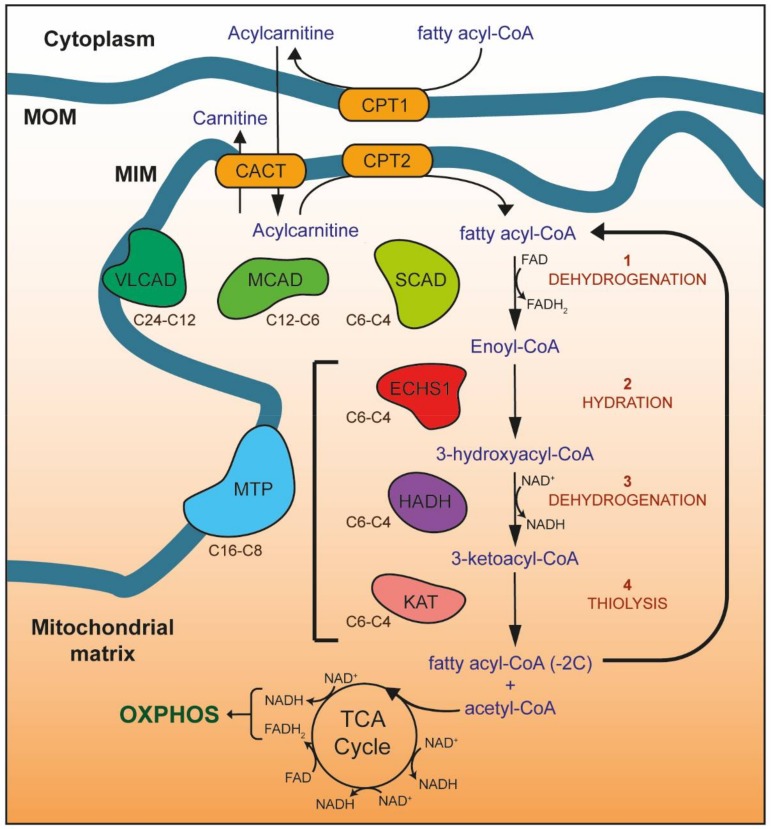
Mitochondrial fatty acid β-oxidation (FAO). Enzymes of the carnitine shuttle system (yellow) are responsible for transporting fatty acyl-CoA esters into the mitochondrial matrix as acylcarnitines. Carnitine is added to fatty acyl-CoAs by carnitine *O*-palmitoyltransferase 1 (CPT1), forming acylcarnitines that are transported into the mitochondrial matrix by the carnitine acylcarnitine translocase (CACT). Once inside the mitochondrial matrix, carnitine *O*-palmitoyltransferase 2 (CPT2) removes the carnitine to regenerate the fatty acyl-CoA ester. Four reactions (**1**–**4**) then occur for each round of FAO, catalyzed by enzymes with different carbon chain length specificities (as shown): **1**—dehydrogenation of fatty acyl-CoA esters by very long-chain (VLCAD), medium-chain (MCAD), and short-chain (SCAD) acyl-CoA dehydrogenases (shown in green) to form enoyl-CoA, **2**—hydration of enoyl-CoA by the mitochondrial trifunctional protein (MTP, blue) or short-chain enoyl-CoA hydratase (ECHS1, red) to form 3-hydroxyacyl-CoA, **3**—dehydrogenation of 3-hydroxyacyl-CoA by MTP or hydroxyacyl-CoA dehydrogenase (HADH, purple) to form 3-ketoacyl-CoA, **4**—thiolysis of 3-ketoacyl-CoA by MTP or 3-ketoacyl-CoA thiolase (KAT, pink). The resulting fatty acyl-CoA is shortened by two carbons, with the generation of acetyl-CoA, NADH and FADH_2_. NADH and FADH_2_ provide electrons for OXPHOS, while acetyl-CoA enters the TCA cycle to generate further NADH and FADH_2_. The shortened fatty acyl-CoA undergoes further rounds of FAO until only two acetyl-CoA molecules remain. MOM, mitochondrial outer membrane; MIM, mitochondrial inner membrane.

Fatty acids are the main energy source during fasting when glucose is not available, and the preferred substrates for catabolic metabolism in the heart, liver and skeletal muscle [6]. Free fatty acids are activated in the cytosol by acyl-CoA synthetases to form fatty acyl-CoA esters. These are subsequently transported into the mitochondria via the carnitine shuttle system. Carnitine *O*-palmitoyltransferase 1 (CPT1) catalyzes the addition of carnitine to fatty acyl-CoA esters to form acylcarnitines, which are transported across the IMM via carnitine acylcarnitine translocase (CACT). Once inside the mitochondrial matrix, carnitine is removed by carnitine *O*-palmitoyltransferase 2 (CPT2) to regenerate fatty acyl-CoA esters and free carnitine, which is recycled back across the IMM by CACT (Figure 1).

Through a series of four enzymatic reactions, dehydrogenation, hydration, a second dehydrogenation and thiolysis, fatty acyl-CoA chains within the mitochondria are processed to yield one acetyl-CoA molecule, two electrons and a fatty acyl-CoA shortened by two carbons. This series of reactions is then performed repeatedly until only two acetyl-CoA molecules remain (commonly termed the β-oxidation spiral) (Figure 1). Both dehydrogenation steps are cofactor-dependent, resulting in the reduction of NAD^+^ and FAD to NADH and FADH_2_, which are subsequently oxidized by OXPHOS complexes I and II respectively [7].

The enzymes involved in FAO exhibit chain length specificity [8]. Very long-, medium-, and short-chain acyl-CoA dehydrogenases (VLCAD, MCAD, SCAD) catalyze the first dehydrogenation step of C24-C12, C12-C6 and C6-C4 carbon chain length fatty acyl-CoAs respectively. For the remaining three reactions, longer acyl-CoA chains (C16-C8) are catalyzed by the multi-domain mitochondrial trifunctional protein (MTP), which harbors long-chain enoyl-CoA hydratase, long-chain 3-hydroxyacyl-CoA dehydrogenase and 3-ketoacyl-CoA thiolase activities [9]. For medium- and short-chain fatty acids, the last three steps of FAO are catalyzed by short-chain enoyl-CoA hydratase (ECHS1), hydroxyacyl-CoA dehydrogenase (HADH) and 3-ketoacyl-CoA thiolase (KAT) (Figure 1).

## 3. ECHS1 is a Multifunctional Enzyme

Short-chain enoyl-CoA hydratase (ECHS1; EC 4.2.1.17) is responsible for the second step of FAO. ECHS1 activity was first observed by Stern and Del Campillo [10] in ox heart and liver, with human ECHS1 cDNA clones first isolated in 1993 [10]. The 11 kb *ECHS1* gene locus was subsequently mapped to chromosome 10q26.2–q26.3 by fluorescence in situ hybridization, encoding eight exons with the 5′ and 3′ untranslated regions contained within exons I and VIII respectively [11].

**Figure 2:**
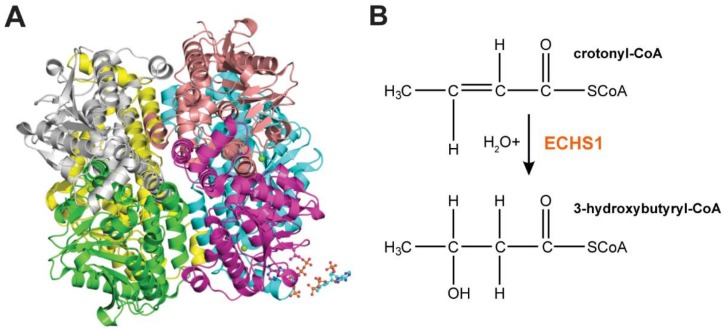
ECHS1 Structure and Function. (**A**) Homohexameric ECHS1 crystal structure at 2.55 Å resolution (PDB: 2hw5), showing six ECHS1 units colored by chain. Two copies of the 4-carbon substrate crotonyl-CoA are shown (bottom right hand corner). (**B**) ECHS1 catalyzes the conversion of trans-Δ^2^-enoyl-CoA thioesters to 3-l-hydroxyacyl-CoA thioesters by stereospecific hydration of the trans double bond between carbons two and three. Hydration of crotonyl-CoA to 3-hydroxybutyryl-CoA is shown.

*ECHS1* is transcribed as a single 1.4 kb mRNA, with expression observed in hepatocytes, fibroblasts and myocytes [10]. The translated 290 amino acid precursor protein contains a 27-amino-acid N-terminal mitochondrial targeting signal that is cleaved upon entry into the mitochondrial matrix [12], where the resulting 28.3-kDa mature protein forms an active 188 kDa homohexamer composed of a ‘dimer of trimers’ [13,14] (Figure 2A).

ECHS1 catalyzes the conversion of trans-Δ^2^-enoyl-CoA thioesters to 3-l-hydroxyacyl-CoA thioesters by stereospecific hydration of the trans double bond between carbons two and three [15] (Figure 2B). ECHS1 has strongest substrate affinity for the 4-carbon crotonyl-CoA, but can bind enoyl-CoA chains up to 10 carbon atoms in length [14]. While ECHS1 has considerably higher specificity for straight-chain enoyl-CoA thioesters as part of the FAO pathway, it also exhibits moderate activity for degrading methacrylyl-CoA (valine pathway), 3-methylcronytyl-CoA (leucine pathway) and tiglyl-CoA (isoleucine pathway) [16,17]. Interestingly, only metabolites of the valine pathway have been detected in plasma and urine of patients with ECHS1 deficiency (ECHS1D), suggesting that ECHS1 is vital for valine metabolism, but not leucine or isoleucine metabolism [16,17].

## 4. FAO Disease

Defects in FAO were first described in the 1970′s in patients with carnitine *O*-palmitoyltransferase deficiencies [18,19,20], with the first pathogenic mutations identified in *ACADM* (which encodes the medium-chain acyl-CoA dehydrogenase) [21,22,23]. Pathogenic mutations have now been identified in at least 22 different FAO genes, and can affect up to 1 in 10,000 individuals in certain populations [1]. Patients can present in early childhood with severe (often lethal) liver dysfunction, hypoglycemia and Reye-like syndrome (a combination of encephalopathy due to acute brain swelling and liver dysfunction caused by fat accumulation) [24]. Cardiac symptoms, such as dilated hypertrophic cardiomyopathy and arrhythmias, are also common [25,26]. Alternatively, ‘milder’ adult-onset disease, presenting with exercise-induced myopathy and rhabdomyolysis (breakdown of muscle fibers), has also been reported [27].

Disease presentation may not be persistent, with patients showing no symptoms or biochemical deficiencies until an episode of metabolic crisis. These episodes can be triggered by various circumstances, including prolonged fasting, exercise, infection, exposure to cold, or a fat-rich diet [7,28,29]. Treatment options are limited, focusing on restricting dietary long-chain fatty acids that cannot be metabolized (and which become toxic), as well as maintaining blood glucose levels.

FAO deficiencies are also believed to cause about 1–3% of unexplained sudden infant deaths [30], with deficiencies in MCAD and long-chain 3-hydroxyacyl-CoA dehydrogenase (LCHAD) reported [31,32]. In addition, mutations in the genes encoding MCAD, SCAD, LCHAD and CPT2 are associated with acute fatty liver of pregnancy (AFLP) and hemolysis, elevated liver enzymes and low platelets (HELLP) syndrome, both of which carry significant neonatal and maternal morbidity and mortality during pregnancy [33,34].

## 5. ECHS1 Deficiency (ECHS1D)

ECHS1D onset is usually at birth or in early childhood, with death occurring within the first two days of life in some cases [35]. The clinical presentation of ECHS1D is typified by Leigh syndrome (subacute necrotizing encephalomyelopathy) or Leigh-like syndrome, with symptoms including (but not limited to) developmental delay, dystonia, metabolic acidosis, cardiomyopathy and apnea. Leigh syndrome is a progressive neurodegenerative disease characterized by bilateral symmetric brain lesions and psychomotor regression [36], and is not typically observed in other FAO disorders [37]. Leigh syndrome has been associated with more than 75 genes, mostly involved in OXPHOS complex I structure and assembly [38]. Brain magnetic resonance imaging (MRI) findings have revealed T_2_ bilateral hyperintensities, a hallmark of Leigh syndrome, in almost all reported cases of ECHS1D. Despite this, there are disparities between the clinical presentation in ECHS1D and the classical features of Leigh syndrome (as defined in Rahman, et al. [39]). Indeed, Haack, et al. [40] proposed that ECHS1D is a distinct form of Leigh-like syndrome associated with severe progressive encephalopathy, accompanied by bilateral brain lesions and mitochondrial dysfunction.

More recently, three patients were identified with clinical symptoms that expand the phenotypic spectrum of ECHS1D [41,42,43]. One patient harboring *ECHS1* variants previously associated with Leigh-like syndrome also presented with cutis laxa, a connective tissue disorder characterized by loose, inelastic skin [43]. Another two patients displayed symptoms of paroxysmal exercise-induced dyskinesia (PED) [41,42]. PED is a much milder form of ECHS1D that offers a more optimistic prognosis, being characterized by recurrent attacks of abnormal dystonic movement that is triggered by prolonged exercise [44]. While these PED patients were unrelated, they shared a common *ECHS1* mutation (c.518C > T; p.Ala173Val), suggesting an association between this variant and PED. Interestingly, T_2_ hyperintensities were still observed in these patients, but Leigh-like symptoms were absent. Conversely, a sibling of one of these patients did suffer from Leigh-like syndrome with severe generalized dystonia, further exemplifying the substantial clinical heterogeneity of ECHS1D [42].

## 6. Pathogenic Mutations in *ECHS1*

Forty-two patients (from 33 families) with ECHS1D have been described to date (Table 1). As with almost all primary disorders of FAO, ECHS1D follows an autosomal recessive pattern of inheritance. Human mutations in *ECHS1* were first described in two infant siblings [45], with all subsequent cases exhibiting compound heterozygosity or homozygosity due to consanguinity. Twenty-seven of the 30 different *ECHS1* mutations identified are missense, suggesting that null mutations may be incompatible with life [40,46]. Other noteworthy variants include nonsense mutations, frameshift mutations, and duplications resulting in protein truncation [35,40,42]. The only incidence of homozygosity for a truncating mutation was reported in two siblings with a very severe phenotype and death within 48 h, establishing some evidence for a genotype-phenotype relationship [35].

While most *ECHS1* mutations are novel, the c.476A > G; p.Gln159Arg variant has been identified in seven unrelated ECHS1D patients with diverse racial backgrounds [17,40,43,47,48]. Furthermore, this variant may also represent a common founder mutation, as it has been independently reported in two families of Pakistani origin [40,48]. A second possible founder mutation in *ECHS1* (c.538A > G; p.Thr180Ala) has also been identified in four French-Canadian patients [47]. Interestingly, this mutation has also been identified in an Irish Traveler family with a haplotype shared with the French-Canadian patients, suggesting an Irish ancestral origin with subsequent migration to Canada [48].

## 7. Biochemical and Metabolic Characterization of ECHS1D

ECHS1 may play a redundant role in FAO, as exemplified by the unremarkable acylcarnitine profiles in most patient cells [16,17,35,40,47,48,49,50,52]. However, extremely high acylcarnitine levels (of C6 and C4 chain lengths) were reported in one patient who died within one day of birth [46]. As such, elevated acylcarnitines may act as an indicator of disease in only the most severe cases of ECHS1D.

Loss of ECHS1 activity for valine metabolism also appears to play a role in disease pathology. ECHS1 acts on both methacrylyl-CoA from the valine pathway and acryloyl-CoA from an alternate pathway of odd-chain FAO that feeds into the valine pathway [54]. In ECHS1D, these two intermediates accumulate, becoming toxic by spontaneous reaction with thiol groups and other mitochondrial cysteine residues, resulting in impaired ATP production and metabolic acidosis [55]. Specifically, methacrylyl-CoA and acryloyl-CoA react with lipoyl domains of the E2 subunit of the pyruvate dehydrogenase complex (PDC), inhibiting its function [17]. Indeed, reduced PDC activity has been observed in many ECHS1D patients, explaining the commonly observed symptom of lactic acidosis [17,35,40,43,45,47,48,51,52]. Notably, an inhibitory effect on two other lipoyl domain-containing mitochondrial enzymes was not detected [17], suggesting inhibition is specific to the PDC (via an unknown mechanism).

These findings have led to tentative correlations between ECHS1D severity, degree of lactic acidosis and reduced PDC activity. In cases where ECHS1D caused early death, lactate levels were high and PDC activity low [35]. Conversely, in mild cases of ECHS1D, lactic acidosis was absent [16,41,42].

Other metabolic markers of ECHS1D, such as increased metabolites of methacrylyl-CoA and acryloyl-CoA in patient urine, have also been identified [45]. In particular, large peaks of 2-methyl-2,3-dihydroxybutyrate have been detected in many ECHS1D cases [40,45,48,50,51]. While the origin of 2-methyl-2,3-dihydroxybutyrate is not known, evidence suggests it is a derivative of acryloyl-CoA [45]. Despite these findings, 2-methyl-2,3-dihydroxybutyrate concentrations have repeatedly measured within the normal range in milder ECHS1D cases [16,17,40,42]. Conversely, *N*-acetyl-*S*-(2-carboxypropyl)cysteine (produced from methacrylyl-CoA) is the only known metabolite that is elevated in the mildest cases of ECHS1D, making it the most useful biomarker for ECHS1D diagnosis [16,42,52].

## 8. Secondary OXPHOS Defects in ECHS1D

FAO and OXPHOS are tightly linked biochemically, with the oxidation of fatty acids generating NADH and FADH_2_ for oxidation by OXPHOS complexes I and II. In addition, studies have also reported physical interactions between FAO and OXPHOS proteins: OXPHOS complex I can bind the FAO protein LCHAD [56], while OXPHOS complex III can be purified in complexes with the FAO electron transfer flavoprotein (ETF) [57]. More recently, the FAO proteins VLCAD, MCAD, LCHAD, and ETF were shown to co-migrate with the OXPHOS supercomplex in a metabolically active super-structure that can oxidize fatty acids [58].

Interestingly, patients with primary deficiencies in LCHAD can exhibit significant OXPHOS enzyme defects [26,59]. These secondary OXPHOS defects were historically attributed to the accumulation of inhibitory fatty-acyl CoA intermediates. However, it now appears that more complex mechanisms are involved. Indeed, we recently showed that loss of the FAO protein MCAD can disrupt OXPHOS complex assembly and stability, resulting in reduced mitochondrial respiration and increased ROS generation in the presence of OXPHOS inhibitors [60].

Secondary OXPHOS defects have also been identified in ECHS1D. These range from isolated defects in complex I, complex III or complex IV to multiple complex I/III/IV or complex I/IV/V defects [40,47,48,49,53]. Furthermore, blue native polyacrylamide gel electrophoresis (BN-PAGE) has identified reduced steady-state levels of mature complex IV in one patient [47]. Conversely, no differences in complex IV levels were detected in three other ECHS1D patients [17,49].

While it is not clear what causes these secondary OXPHOS defects in ECHS1D, disruption of complex I activity may be explained via its interaction with the pyruvate dehydrogenase complex (PDC). As discussed above, toxic metabolites of the valine pathway can accumulate in ECHS1D and inhibit PDC activity [17]. This inhibition may disrupt the binding of complex I to the PDC [56], with a potential loss of both complex I stability and NADH dehydrogenase activity.

Overall, it is difficult to predict the effect of ECHS1D on OXPHOS. However, it is apparent that patients with secondary OXPHOS defects generally exhibit more severe Leigh-like symptoms (Table 1).

## 9. Concluding Remarks

Forty patients have been described with pathogenic mutations in *ECHS1* since the identification of the first two patients with ECHS1D in 2014. Notably, most ECHS1D patients present with Leigh syndrome or Leigh-like syndrome, a severe disorder traditionally associated with defects in OXPHOS complex I activity. While loss of ECHS1 disrupts both FAO and valine metabolism, secondary OXPHOS defects have also been identified in some patients with ECHS1D. Furthermore, these secondary OXPHOS defects are associated with a more severe clinical phenotype, suggesting that they also contribute to disease pathology.

Like other FAO deficiencies, secondary OXPHOS defects in ECHS1D may be due to the accumulation of toxic fatty acid and/or valine metabolites, which can directly inhibit OXPHOS complex activity. Alternatively, the effects of these metabolites may be indirect by disrupting the interaction between the pyruvate dehydrogenase complex (PDC) and OXPHOS complex I. Whichever mechanism is involved, further research is required to clarify the relationship between ECHS1D and OXPHOS dysfunction, and to determine if the stability and/or biogenesis of the OXPHOS complexes is also disrupted by the loss of ECHS1. This knowledge will be invaluable for our understanding of the complex interactions between the FAO and OXPHOS pathways, and will help to advance the diagnosis and treatment of mitochondrial disorders such as ECHS1D.

## Figures and Tables

**Table 1:** The clinical, biochemical and metabolic features of all reported ECHS1D patients.

**Reference**	**Peters et al. 2014 [45]**		**Sakai et al. 2015 [49]**	**Haack et al. 2015 [40]**				
**Patient ID**	Patient 1	Patient 2	Patient 1	FI, II:2	F2: II:1	F3. II:6	F4; II:1	
**Age at presentation**	Birth	3 months	2 months	Birth	Birth	Birth	Birth	
**Death**	4 months	8 months	NL	4 months	11 months	3 years	7.5 years	
**Parental consanguinity**	No	No	No	No	No	Yes	No	
**Mutation 1 (genetic level; protein effect)**	c.473C > A; p.Ala158Asp	c.473C > A; p.Ala158Asp	c.2T > G; p.Met1Arg	c.176A > G; p.Asn59Ser	c.197T > C; p.Ile66Thr	c.476A > G; p.Gln159Arg	c.161G > A; p.Arg54His	
**Mutation 2 (genetic level; protein effect)**	c.414 + 3G > C; splicing	c.414 + 3G > C; splicing	c.5C > T; p.Ala2Val	c.476A > G; p.Gln159Arg	c.449A > G; p.Asp150Gly	c.476A > G; p.Gln159Arg	c.817A > G; p.Lys273Glu	
**T_2_ hyperintensity**	Yes	NL	Yes	Yes	Yes	Yes	NL	
**Acylcarnitine profile**	ND	ND	Normal	Normal	Normal	Normal	NL	
**PDC activity**	Reduced	Reduced	ND	ND	Reduced	ND	ND	
**OXPHOS activity**	ND	ND	Reduced CI, CIII and CIV (patient cells), reduced CI, CIV and CV (immortalized myoblasts)	Reduced CI in liver, normal in heart and muscle	Normal	ND	Normal (but reduced overall ATP production)	
**OXPHOS complex steady-state levels**	ND	ND	Normal	ND	ND	ND	ND	
**Reference**		**Haack et al. 2015 [40] (continued)**						
**Patient ID**	F5; II:3	F6, II:1	F7, II:2	F8, II:1	F9, II:2	F10, II:1		
**Age at presentation**	Birth	Birth	2 years	1 year	Birth	11 months		
**Death**	Alive at 2.3 years	Alive at 3 years	Alive at 5 years	Alive at 8 years	Alive at 16 years	Alive at 31 years		
**Parental consanguinity**	Yes	No	No	No	No	No		
**Mutation 1 (genetic level; protein effect)**	c.673T > C; p.Cys225Arg	c.98T > C; p.Phe33Ser	c.268G > A, p.Gly90Arg	c.161G > A; p.Arg54His	c.161G > A; p.Arg54His	c.229G > C; p.Glu77Gln		
**Mutation 2 (genetic level; protein effect)**	c.673T > C; p.Cys225Arg	c.176A > G; p.Asn59Ser	c.583G > A; p.Gly195Ser	c.394G > A; p.Ala132Thr	c.431dup; p.Leu145Alafs*6	c.476A > G; p.Gln159Arg		
**T_2_ hyperintensity**	Yes	Yes	Yes	ND	Yes	Yes		
**Acylcarnitine profile**	Normal	Normal	NL	NL	NL	NL		
**PDC activity**	ND	ND	ND	ND	Normal	ND		
**OXPHOS activity**	Normal	Reduced CIV in muscle	Normal	ND	Normal	Normal		
**OXPHOS complex steady-state levels**	ND	ND	ND	ND	ND	ND		
**Reference**	**Ferdinandusse et al. 2015** [17]				**Tetreault et al. 2015** [47]			
**Patient ID**	Patient 1	Patient 2	Patient 3	Patient 4	P1	P2	P3	P4
**Age at presentation**	Birth	Birth	Early infancy	1 year	2.5 months	2.9 years	10 months	6 months
**Death**	24 h	2 days	Alive at 7 years	3 years	10 months	Alive at 18 years	Alive at 13 years	Alive at 12 years
**Parental consanguinity**	Yes	Yes	No	No	No	No	No	No
**Mutation 1 (genetic level; protein effect)**	c.817A > G; p.Lys273Glu	c.817 > G; p.Lys273Glu	c.433C > T; p.Leu145Phe	c.673T > C; p.Cys225Arg	c.538A > G; p.Thr180Ala	c.538A > G; p.Thr180Ala	c.538A > G; p.Thr180Ala	c.538A > G; p.Thr180Ala
**Mutation 2 (genetic level; protein effect)**	c.817A > G; p.Lys273Glu	c.817A > G; p.Lys273Glu	c.476A > G; p.Gln159Arg	c.674G > C; p.Cys225Ser	c.583G > A; p.Gly195Ser	c.713C > T; p.Ala238Val	c.713C > T; p.Ala238Val	c.476A > G; p.Gln159Arg
**T_2_ hyperintensity**	ND	Yes	NL	Yes	Yes	Yes	Yes	Yes
**Acylcarnitine profile**	Normal	Normal	Normal	Normal	Normal	Normal	Normal	Normal
**PDC activity**	Reduced	Reduced	ND	ND	Reduced	Normal	ND	Normal
**OXPHOS activity**	Normal	Normal	ND	ND	Mild reduction of CI and CIII in muscle	Normal	Normal	Normal
**OXPHOS complex steady-state levels**	Normal	Normal	ND	ND	ND	ND	ND	Reduced CIV in fibroblasts
**Reference**	**Yamada et al. 2015** [16]		**Ganetzky et al. 2016** [50]		**Olgiati et al. 2016** [42]		**Nair et al. 2016** [46]	
**Patient ID**	III-2	III-3	Patient 1	Patient 2	II-1	II-2	Patient 1	
**Age at presentation**	10 months	7 months	Prenatal	Prenatal	3.5 years	4.5 years	Birth	
**Death**	Alive at 7 years	5 years	16 h	24 h	Alive at 17 years	Alive at 15 years	24 h	
**Parental consanguinity**	No	No	No	No	No	No	Yes	
**Mutation 1 (genetic level; protein effect)**	c.176A > G; p.Asn59Ser	c.176A > G; p.Asn59Ser	c.8C > A; p.Ala3Asp	c.8C > A; p.Ala3Asp	c.232G > T; p.Glu78Ter	c.232G > T; p.Glu78Ter	c.842A > G; p.Glu281Gly	
**Mutation 2 (genetic level; protein effect)**	c.413C > T; p.Ala138Val	c.413C > T; p.Ala138Val	c.389T > A; p.Val130Asp	c.389T > A; p.Val130Asp	c.518C > T; p.Ala173Val	c.518C > T; p.Ala173Val	c.842A > G; p.Glu281Gly	
**T_2_ hyperintensity**	Yes	Yes	ND	ND	Yes	Yes	ND	
**Acylcarnitine profile**	Normal	Normal	Mild C4 elevation	Mild C4 elevation	ND	ND	Elevated C4 and C6	
**PDC activity**	ND	ND	ND	ND	ND	ND	ND	
**OXPHOS activity**	Normal	ND	ND	ND	ND	ND	ND	
**OXPHOS complex steady-state levels**	ND	ND	ND	ND	ND	ND	ND	
**Reference**	**Mahajan et al. 2017** [41]	**Al Mutairi et al. 2017** [35]		**Balasubramaniam et al. 2017** [43]		**Bedoyan et al. 2017** [51]		**Huffnagel et al. 2017** [52]
**Patient ID**	Patient 1	Patient 1	Patient 2	Patient 1		Patient 1		Patient 1
**Age at presentation**	8 years	Birth	Birth	17 months		Birth		6 weeks
**Death**	Alive at 8 years	2 days	8 ho	Alive at 4.5 years		39 days		Alive at 26 years
**Parental consanguinity**	No	Yes	Yes	No		No		No
**Mutation 1 (genetic level; protein effect)**	c.518C > T; p.Ala173Val	c.88 + 5G > A; p.Ala31Glufs*23	c.88 + 5G > A; p.Ala31Glufs*23	c.476A > G; p.Gln159Arg		c.836T > C; p.Phe279Ser		c.229G > C p.Glu77Gln
**Mutation 2 (genetic level; protein effect)**	c.817A > G; p.Lys273Glu	c.88 + 5G > A; p.Ala31Glufs*23	c.88 + 5G > A; p.Ala31Glufs*23	c.538A > G; p.Thr180Ala		c.8C > A; p.Ala3Asp		c.563C > T p.Ala188Val
**T_2_ hyperintensity**	Yes	ND	ND	Yes		Yes		Yes
**Acylcarnitine profile**	ND	Mild C3, C4, C5 and C10 elevation	Normal	ND		ND		Normal
**PDC activity**	ND	Reduced	Normal	ND		Reduced		ND
**OXPHOS activity**	ND	ND	Normal	ND		Reduced		Normal
**OXPHOS complex steady-state levels**	ND	ND	ND	ND		ND		ND
**Reference**	**Ogawa et al. 2017** [53]				**Fitzsimons et al. 2018** [48]			
**Patient ID**	Pt376	Pt536	Pt1038	Pt1135	Patient 1	Patient 2	Patient 3	Patient 4
**Age at presentation**	NL	NL	NL	NL	5 months	3 months	5 months	2 weeks
**Death**	NL	NL	NL	NL	3 years	21 months	28 months	13 months
**Parental consanguinity**	No	No	No	No	Yes	Yes	Yes	Yes
**Mutation 1 (genetic level; protein effect)**	c.98T > C; p.Phe33Ser	c.5C > T; p.Ala2Val	c.5C > T; p.Ala2Val	c.5C > T; p.Ala2Val	c.476A > G; p.Gln159Arg	c.538A > G; p.Thr180Ala	c.538A > G; p.Thr180Ala	c.538A > G; p.Thr180Ala
**Mutation 2 (genetic level; protein effect)**	c.176A > G; p.Asn59Ser	c.1A > G; p.Met1Val	c.176A > G; p.Asn59Ser	c.176A > G; p.Asn59Ser	c.476A > G; p.Gln159Arg	c.538A > G; p.Thr180Ala	c.538A > G; p.Thr180Ala	c.538A > G; p.Thr180Ala
**T_2_ hyperintensity**	ND	ND	ND	ND	Yes	Yes	Yes	Yes
**Acylcarnitine profile**	ND	ND	ND	ND	Normal	ND	Mild reduction of free carnitine and long-chain acylcarnitines	Normal
**PDC activity**	ND	ND	ND	ND	Reduced	ND	Normal	ND
**OXPHOS activity**	Reduced CIV	Normal	Normal (but reduced oxygen consumption rate)	Reduced CI	Normal	ND	Reduced CIII in muscle	ND
**OXPHOS complex steady-state levels**	ND	ND	ND	ND	ND	ND	ND	ND

T_2_ hyperintensity refers to regions of high intensity on T_2_ weighted magnetic resonance imaging scans of the brain. OXPHOS complex steady-state levels were determined by blue native polyacrylamide gel electrophoresis. PDC, pyruvate dehydrogenase complex; OXPHOS, oxidative phosphorylation; ND, not determined; NL, not listed; CI, complex I; CIII, complex III; CIV, complex IV; CV, complex V; C3DC, malonylcarnitine; C4, butyrylcarnitine; C5DC, glutarylcarnitine; C6, hexanoylcarnitine; C10, decanoylcarnitine.
